# Blood-Based Biomarkers to Search for Atrial Fibrillation in High-Risk Asymptomatic Individuals and Cryptogenic Stroke Patients

**DOI:** 10.3389/fcvm.2022.908053

**Published:** 2022-07-04

**Authors:** Elena Palà, Alejandro Bustamante, Jorge Pagola, Jesus Juega, Jaume Francisco-Pascual, Anna Penalba, Maite Rodriguez, Mercedes De Lera Alfonso, Juan F. Arenillas, Juan Antonio Cabezas, Soledad Pérez-Sánchez, Francisco Moniche, Reyes de Torres, Teresa González-Alujas, Josep Lluís Clúa-Espuny, Juan Ballesta-Ors, Domingo Ribas, Juan Acosta, Alonso Pedrote, Felipe Gonzalez-Loyola, Delicia Gentile Lorente, Miguel Ángel Muñoz, Carlos A. Molina, Joan Montaner

**Affiliations:** ^1^Neurovascular Research Laboratory, Vall d’Hebron Institute of Research, Hospital Vall d’Hebron, Universitat Autònoma de Barcelona, Barcelona, Spain; ^2^Stroke Unit, Hospital Universitari Germans Trias i Pujol, Badalona, Spain; ^3^Stroke Unit, Medicine Department, Vall d’Hebrón Hospital and Autonomous University of Barcelona, Barcelona, Spain; ^4^Arrhythmia Unit-Cardiology Department, Vall d’Hebrón Hospital, Barcelona, Spain; ^5^Centro de Investigación Biomédica en Red de Enfermedades Cardiovasculares (CIBER-CV), Madrid, Spain; ^6^Stroke Unit, University Hospital of Valladolid, Valladolid, Spain; ^7^Stroke Unit, University Hospital Virgen del Rocio, Seville, Spain; ^8^Stroke Unit, University Hospital Virgen Macarena, Seville, Spain; ^9^Echocardiography Lab Cardiology Department, Vall d’Hebrón Hospital, Barcelona, Spain; ^10^Equip d’Atenció Primària Tortosa Est, SAP Terres de l’Ebre, Institut Català de la Salut, Tortosa, Spain; ^11^Institut d’Investigació en Atenció Primària IDIAP Jordi Gol, Ebrictus Group, Barcelona, Spain; ^12^EAP Sant Pere i Sant Pau, DAP Camp de Tarragona, Institut Català de la Salut, Tarragona, Spain; ^13^Department of Cardiology, Hospital Universitario Virgen del Rocio, Seville, Spain; ^14^Gerència Atenció Primària de Barcelona, Institut Català de la Salut, Barcelona, Spain; ^15^Institut d’Investigació en Atenció Primària IDIAP Jordi Gol, Unitat Suport Recerca Barcelona, Barcelona, Spain; ^16^Cardiology Department, Hospital Verge de la Cinta, Institut Català de la Salut, Tortosa, Spain

**Keywords:** atrial fibrillation, stroke, biomarkers, screening, cryptogenic stroke

## Abstract

**Background:**

Atrial fibrillation (AF) increases the risk of ischemic stroke in asymptomatic individuals and may be the underlying cause of many cryptogenic strokes. We aimed to test the usefulness of candidate blood-biomarkers related to AF pathophysiology in two prospective cohorts representative of those populations.

**Methods:**

Two hundred seventy-four subjects aged 65–75 years with hypertension and diabetes from the AFRICAT cohort, and 218 cryptogenic stroke patients aged >55 years from the CRYPTO-AF cohort were analyzed. AF was assessed by 4 weeks of monitoring with a wearable Holter device (NuuboTM™). Blood was collected immediately before monitoring started. 10 candidate biomarkers were measured by automated immunoassays (Roche, Penzberg) in the plasma of all patients. Univariate and logistic regression analyses were performed in each cohort separately.

**Results:**

Atrial fibrillation detection rate was 12.4% (AFRICAT cohort) and 22.9% (CRYPTO-AF cohort). 4 biomarkers were significantly increased in asymptomatic individuals with AF [Troponin-T, Angiopoietin-2 (Ang-2), Endocan, and total N-terminal pro-B type natriuretic peptide (NT-proBNP)] and 7 biomarkers showed significantly higher concentrations in cryptogenic stroke patients with AF detection [growth differentiation factor 15, interleukin 6, Troponin-T, Ang-2, Bone morphogenic protein 10, Dickkopf-related protein 3 (DKK-3), and total NT-proBNP]. The models including Ang-2 and total NT-proBNP [AUC 0.764 (0.665–0.863)], and Ang-2 and DKK-3 [AUC = 0.733 (0.654–0.813)], together with age and sex, showed the best performance to detect AF in high-risk asymptomatic individuals, and in cryptogenic stroke patients, respectively.

**Conclusion:**

Blood-biomarkers, in particular, total NT-proBNP, DKK-3, and Ang-2, were associated with AF reflecting two mechanistically different pathways involved in AF pathophysiology (AF stretch and vascular changes). The combination of these biomarkers could be useful in AF screening strategies in the primary care setting and also for searching AF after cryptogenic stroke.

## Introduction

Atrial fibrillation (AF) increases the risk of ischemic stroke in asymptomatic individuals and may be the underlying cause of many cryptogenic strokes (1). Anticoagulant therapy is the most effective treatment to reduce stroke risk in the presence of AF (2), but this arrhythmia is usually underdiagnosed and, therefore, undertreated. Pre- and post-stroke AF searching would increase AF detection, and subsequently the number of patients that would benefit from primary and secondary stroke prevention treatments (3, 4).

Some proteins increase in AF individuals and may be useful as AF biomarkers. Until now, natriuretic peptides [B-type natriuretic peptide (BNP) and N-terminal pro-B type natriuretic peptide (NT-proBNP)] (5, 6) are some of the most promising candidates, given their high expression in the atrium under pressure (7), and its correlation with atrial enlargement observed in AF (8). However, the behavior of natriuretic peptides as biomarkers is not perfect and could be complemented by others representative of pathways involved in the AF pathophysiology. AF pathophysiology has been described to include atrial and vascular changes, such as inflammation, myocyte injury, collagen, and lipid infiltration. Therefore, interleukin 6 (IL-6), Troponin-T, growth differentiation factor 15 (GDF-15), and fibroblast growth factor 23 (FGF-23), markers of inflammation, myocardial damage, oxidative stress, and atrial fibrosis, respectively, have been extensively associated with AF occurrence, recurrence, and/or AF prognosis (9).

There are other proteins, that althought the literature concerning AF is scarcer, are interesting candidates to be further tested. Endocan (ESM-1) is a marker of inflammation and endothelial dysfunction proposed as a new biomarker for the prediction of stroke risk among patients with AF (10). Bone morphogenic protein 10 (BMP-10) is an atrial-specific protein that plays an important role in heart development and predicts AF recurrence after ablation (11). Angiopoietin-2 (Ang-2) is an endothelial growth factor reported to be increased in AF (12–14). Dickkopf-related protein 3 (DKK-3) is involved in heart development and cardiac hypertrophy protection and, in AF patients, elevated levels have been found in the atrial appendages and in circulation (15, 16). Finally, insulin-like growth factor-binding protein 7 (IGFBP-7) is a marker of myocardial damage that has been independently associated with chronic heart failure in AF patients (17).

**FIGURE 1:**
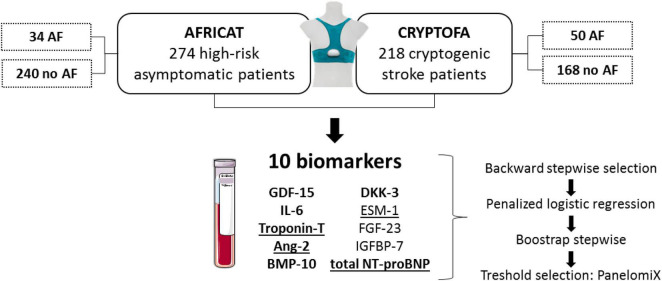
Study design. Patients from two cohorts were included in the present study. In both cohorts, patients were monitored for 28 days to identify paroxysmal AF patients. Biomarkers displayed were measured in all patients. Those underlined had significantly higher concentration in AF patients from the AFRICAT cohort, while the ones in bold had significantly higher concentration in AF patients from the CRYPTOFA cohort. GDF-15 indicates growth differentiation factor 15; IL-6, interleukin 6; Ang-2, angiopoietin 2; BMP-10, bone morphogenic protein 10; DKK-3, dickkopf-related protein 3; ESM-1, endocan or endothelial cell specific molecule-1; FGF-23, fibroblast growth factor 23; IGFBP-7, insulin-like growth factor-binding protein 7; and NT-proBNP, N-terminal pro-brain natriuretic peptide.

In the present study, we aimed to test the performance of candidate blood-biomarkers to detect AF in two prospective cohorts: one with asymptomatic high-risk subjects and the other with cryptogenic stroke patients (Figure 1).

## Materials and Methods

### Subjects

#### AFRICAT Study

Asymptomatic individuals between 67 and 75 years old with hypertension and diabetes were included in the AFRICAT cohort, an observational, prospective, multicenter, population-based screening study for AF (6). Individuals with chronic inflammatory diseases, cancer, or dementia were excluded. AF diagnosis was assessed by a baseline 12-lead electrocardiogram (ECG), 28 days monitoring with a wearable Holter device (Nuubo™), and/or medical history (MH). From the 359 patients included in the cohort, those without blood samples (*n* = 2), and/or short/bad quality Holter registers from the no AF group (*n* = 83) were excluded. In total, 274 individuals were analyzed in the present study. AF burden was calculated from the Holter records as minutes being in AF divided by the total minutes of readable records and was expressed as a percentage. Plasma NT-proBNP measurements were available from all the patients included (6).

The AFRICAT study protocol was approved by the clinical research ethics committees of IDIAP Jordi Gol (P15/047) and Hospital Universitari Vall d’Hebron [PR (AG) 133-2015]. All participants signed informed consent before inclusion. The study protocol conformed to the ethical guidelines of the 1975 Declaration of Helsinki.

#### CRYPTO-AF Study

Cryptogenic ischemic stroke patients (<72 h after stroke) over 55 years of age were included in the CRYPTO-AF cohort (18). All patients underwent complete etiological workup before the cryptogenic stroke classification (ECG, Doppler ultrasound study of the extracranial and intracranial arteries, routine transthoracic echocardiography, and in-hospital ECG continuous automatic monitoring). Patients with known causes of stroke, previous AF, pacemaker carriers, moderate or severe disability after stroke (modified Rankin Scale score > 3), and those with clinical worsening were excluded. Patients were monitored for 28 days to detect AF with a wearable Holter device (Nuubo™) starting within the first 72 h from stroke symptoms. From the 296 patients included in the cohort, 264 completed the Holter monitoring period and 218 had available blood samples which were analyzed in the present study. The number of AF episodes and the longest AF episode in the Holter register were used as AF burden measures in this cohort.

Left atrial volume adjusted to body surface index (LAVI, ml/m^2^) was measured by biplane transthoracic echocardiography in a subset of 152 patients and peak atrial longitudinal strain was evaluated by speckle tracking software (GE EchoPAC^®^) in 131 patients. Plasma NT-proBNP and BNP measurements were available from all the patients included (5).

Written informed consent was obtained from all participants and the study was approved [PR (AG)49/2014] by the Ethical Committee of Vall d’Hebrón Hospital, Valladolid Hospital, Virgen Macarena Hospital and Virgen del Rocio Hospital, in line with Helsinki guidelines.

### Biomarker Quantification

Blood was collected into EDTA tubes at the time of inclusion of each study In the CRYPTO-FA study, blood was drawn post-stroke (<72 h). In the AFRICAT study blood was drawn in the baseline visit together with the performance of the baseline 12-lead ECG. In both studies blood was collected before the AF monitoring with the Holter device started.

After centrifugation at 1,500 *g* and 4^°^C for 15 min, plasma aliquots were frozen at –80^°^C until biomarker determination. Biomarkers were centrally measured and blinded to clinical data (Roche Diagnostics, Penzberg, Germany). GDF-15, IL-6, and Troponin-T were measured with commercial *in vitro* diagnostic immunoassays (Roche Diagnostics). Angiopoietin-2, BMP-10, DKK-3, ESM-1, FGF-23, IGFBP-7, and total NT-proBNP (including both glycosylated and non-glycosylated forms) were quantified using a research grade prototype assay on a Cobas Elecsys e601 platform (Roche Diagnostics GmbH, Penzberg, Germany) employing Elecsys electrochemiluminescence technology. More details on these biomarkers are provided in the Supplementary Table 1.

### Statistical Analysis

Statistical analysis was conducted with SPSS version 21 and R version 3.6.3. Graphs were elaborated with GraphPad Prism 5. Kolmogorov–Smirnov test was used to assess the normality of the data. Data were expressed as number (%) for categorical variables and as mean ± SD or median (interquartile range) for continuous variables, depending on the data distribution. Association between biomarkers and AF diagnosis was analyzed first by univariate analysis. Mann–Whitney *U*-test or Student’s *t*-test were used for continuous variables, and the χ^2^ test was used for categorical variables. ANOVA or the Kruskal–Wallis test were used to compare > 2 variables depending on the variable distribution. The Spearman test was used for correlations.

Samples below the limit of detection were substituted by the lowest value detected minus 0.5 for the analysis. Similarly, samples above the limit of detection were substituted by the highest value detected plus 0.5. Outliers were defined as samples with *Z*-scores of ±3. Outliers were included in the analysis but sensitivity analyses were performed excluding them.

The rms package was used to fit penalized logistic regression models using penalized maximum likelihood estimation. The penalty factor applied in each model was selected to minimize the Hurvich and Tsai’s corrected AIC of the resulting model. Backward stepwise using the AIC criteria, and forcing the inclusion of sex and age as covariates, was used to select the biomarkers to include in the model from all the biomarkers analyzed. Multicollinearity among the predictor variables has been checked with the variance inflation factor. In order to assess the stability of the variable selection process, backward stepwise was applied to 1,000 bootstrap iterations and the most frequent combinations were shown.

The area under the receiving operator curve (AUC) of the constructed models was calculated and compared with a basic clinical model including age and sex using the DeLong test. The integrated discrimination improvement index (IDI) was used to compare their predictive capacity. Also, other cardiovascular risk factors available in each cohort were included in the models in order to avoid influence of comorbidities.

The PanelomiX algorithm (19) was used to select thresholds for the biomarkers identified. PanelomiX uses the iterative combination of biomarkers and thresholds to obtain the combination that provides the optimal classification performance. Two optimization options were used: optimizing global accuracy and optimizing the specificity at ≥90% sensitivity. Again, logistic regression models were created including the panels selected by PanelomiX adjusted by age and sex (including those variables into the model together with the panel).

The analyses for the selection of the best biomarker combinations were carried out separately and independently in each of the cohorts.

The performance of the best biomarker combination was compared to the performance of biomarkers previously measured in the tested cohorts: NT-proBNP (AFRICAT and CRYPTO-AF) and BNP [which had higher accuracy than NT-proBNP in the CRYPTO-AF cohort (5)].

## Results

Baseline characteristics of each cohort and biomarker results were reported in Table 1.

**TABLE 1:** AF univariate analysis of the two included cohorts.

	AFRICAT			CRYPTO-AF		
	AF (*n* = 34)	No AF (*n* = 240)	*P*-value	AF (*n* = 50)	No AF (*n* = 168)	*P*-value
Age	70 (67.5–74)	71 (68.25–73)	0.351	78 (73–83)	73 (67–81)	0.005
Sex (%female)	14 (41.2%)	117 (48.8%)	0.408	25 (50%)	83 (49.7%)	0.970
Hypertension	34 (100%)	240 (100%)	1.00	39 (78%)	128 (77.1%)	0.895
Diabetes	34 (100%)	240 (100%)	1.00	11 (24.4%)	37 (23.4%)	0.886
Ischemic cardiopathy	9 (26.5%)	43 (17.9%)	0.234	3 (6%)	11 (6.5%)	1.000
Heart failure*	3 (8.8%)	13 (5.4%)	0.431	1 (2.1%)	3 (2.5%)	1.000
LAVI	–	–	–	31 (28–39)	27 (23–32.8)	0.002
LAS	–	–	–	21.33 ± 19.69	28.64 ± 9.83	<0.001
BNP (pg/ml)	–	–	–	90.70 (49.87–162.30)	33.65 (14.85–81.87)	<0.001
NT-proBNP (pg/ml)	380.60 (123.97–1,285.50)	112.70 (61.10–206.15)	<0.001	425.95 (226–816.87)	215.90 (99.09–463.85)	<0.001
GDF-15 (pg/ml)	2,367.00 (1,329.25–3,198.75)	2,491 (1,633.5–3,583.00)	0.375	1,896.5 (1,521.5–2,659.5)	1,645.5 (1,169.5–2,356.5)	0.019
IL-6 (pg/ml)	3.64 (2.07–4.91)	3.15 (1.87–4.89)	0.334	11.19 (4.64–20.77)	7.28 (2.84–15.28)	0.042
TroponinT (pg/ml)	16.89 (11.77–23.88)	12.88 (9.73–18.85)	0.011	17.19 (14.27–28.09)	15.11 (10.26–20.95)	0.002
Ang-2 (ng/ml)	2.46 (1.76–4.11)	1.67 (1.37–2.14)	<0.001	2.43 (1.60–3.68)	1.68 (1.32–2.31)	<0.001
BMP-10 (ng/ml)	2.24 (1.84–2.68)	2.06 (1.86–2.31)	0.079	2.26 (1.92–2.53)	2.05 (1.79–2.33)	0.013
DKK3 (ng/ml)	55.84 (51.05–66.29)	54.33 (47.00–66.11)	0.413	62.78 (55.52–79.56)	55.61 (49.37–65.10)	0.001
ESM-1 (pg/ml)	2,087.75 (1,831.47–2,460.95)	1,815.55 (1,482.70–2,297.15)	0.012	2,750.00 (2,025.17–3,280.00)	2,268.80 (1,846.80–3,226.90)	0.101
FGF-23 (pg/ml)	167.73 (129.75–257.84)	148.80 (118.39–206.60)	0.071	134.04 (110.00–204.46)	134.74 (102.11–185.65)	0.583
IGFBP-7 (ng/ml)	115.66 (99.83–132.21)	107.53 (94.91–128.29)	0.088	104.42 (85.50–116.84)	94.68 (82.11–109.56)	0.052
Total NT-proBNP (pg/ml)	1,821.35 (749.58–4,434.00)	650.93 (376.97–1,159.77)	<0.001	2,123.50 (1,241.72–3,555.57)	906.18 (419.09–1,887.40)	<0.001

*Heart failure is considered when LVEF < 40% in th CRYPTO-AF study.

*LAVI indicates left atrial volume index; LAS, left atrial strain.*

**FIGURE 2:**
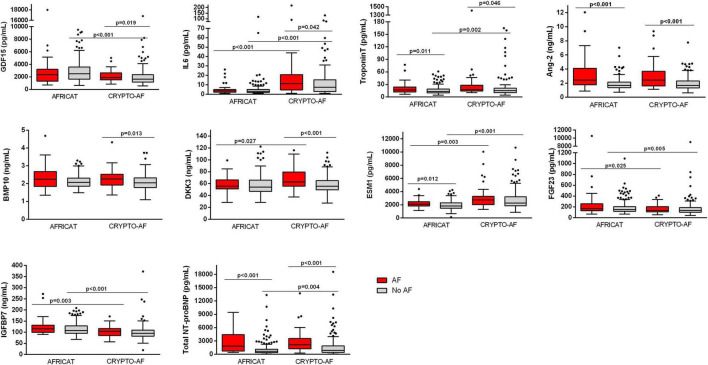
Boxplot distribution of the biomarker circulating levels in the CRYPTO-AF and the AFRICAT cohort. Boxes extend from the 25th to 75th percentiles. The line in the middle is plotted as the median. Whiskers are drawn according to Tukey methodology (±1.5 IQR) and larger values are plotted as individual points. Significant comparisons are indicated with an asterisk.

In the AFRICAT cohort, AF was detected in 34 subjects. AF cases were classified according to how AF diagnosis was performed in 4 groups, defined by medical history (MH) for AF, ECG findings, and Holter AF detection. According to this classification, 17 individuals were only diagnosed during the Holter monitoring period (MH-ECG-Holter+), 7 individuals had a new-onset AF on baseline ECG confirmed during the Holter register (MH-ECG + Holter+), 7 individuals had previous MH of AF, confirmed by ECG in the baseline visit (MH + ECG+), and finally 3 patients had an external diagnosis of AF, previous to the study or during the study, not detected by the ECG or the Holter in the present study (MH + ECG-Holter-). According to this classification, 14 patients had ongoing AF when blood was collected. Among the analyzed molecules, 4 proteins showed significant differences when comparing asymptomatic individuals with and without AF diagnosis (Troponin-T, Ang-2, ESM-1, and total NT-proBNP; Table 1 and Figure 2). None of them showed significant differences when comparing paroxysmal AF cases only detected during the monitoring period (MH-ECG-Holter+) vs no AF cases. Several proteins showed increased levels in the AF groups with “established AF” (MH + ECG + or MH-ECG + Holter+; Supplementary Figure 1). In this cohort, AF burden information was available from 27 patients. The median AF burden was 3% (0.6–14%) and it correlated with FGF-23 (*r* = 0.394, *p* = 0.042), and Total NT-proBNP (*r* = 0.525, *p* = 0.005; Table 2).

**TABLE 2:** Correlations between biomarkers and measures of AF burden and left atrial function.

	AFRICAT	CRYPTO-AF			
	AF burden (%) (*n* = 27)	Number of AF episodes (*n* = 38)	Longest AF episode (minutes; *n* = 38)	LAVI (left atrial volumen index; *n* = 152)	LAS (left atrial strain; *n* = 131)
GDF-15 (pg/ml)	*r* = 0.310, *p* = 0.115	*r* = 0.137, *p* = 0.412	***r* = 0.327, *p* = 0.045**	***r* = 0.185, *p* = 0.023**	***r* = –0.186, *p* = 0.034**
IL-6 (pg/ml)	*r* = –0.136, *p* = 0.498	*r* = 0.105, *p* = 0.529	***r* = 0.379, *p* = 0.019**	*r* = 0.145, *p* = 0.074	***r* = –0.362, *p* < 0.001**
TroponinT (pg/ml)	*r* = 0.359, *p* = 0.072	*r* = 0.054, *p* = 0.430	***r* = 0.344, *p* = 0.034**	***r* = 0.245, *p* = 0.002**	***r* = –0.322, *p* < 0.001**
Ang-2 (ng/ml)	*r* = 0.244, *p* = 0.220	***r* = 0.395, *p* = 0.014**	***r* = 0.518, *p* = 0.001**	*r* = 0.159, *p* = 0.051	***r* = –0.302, *p* < 0.001**
BMP-10 (ng/ml)	*r* = 0.362, *p* = 0.064	*r* = 0.096, *p* = 0.565	*r* = 0.100, *p* = 0.550	***r* = 0.174, *p* = 0.033**	***r* = –0.275, *p* = 0.002**
DKK3 (ng/ml)	*r* = 0.271, *p* = 0.171	*r* = 0.289, *p* = 0.078	*r* = 0.309, *p* = 0.059	***r* = 0.253, *p* = 0.002**	***r* = –0.295, *p* = 0.001**
ESM-1 (pg/ml)	*r* = –0.081, *p* = 0.691	*r* = 0.185, *p* = 0.267	*r* = 0.181, *p* = 0.276	***r* = 0.227, *p* = 0.005**	***r* = –0.221, *p* = 0.012**
FGF-23 (pg/ml)	***r* = 0.394, *p* = 0.042**	*r* = 0.088, *p* = 0.597	*r* = 0.146, *p* = 0.381	*r* = –0.003, *p* = 0.967	*r* = –0.061, *p* = 0.490
IGFBP-7 (ng/ml)	*r* = 0.315, *p* = 0.110	*r* = 0.122, *p* = 0.464	*r* = 0.071, *p* = 0.674	*r* = 0.139, *p* = 0.089	*r* = –0.134, *p* = 0.127
Total NT-proBNP (pg/ml)	***r* = 0.525, *p* = 0.005**	*r* = 0.149, *p* = 0.373	***r* = 0.376, *p* = 0.020**	***r* = 0.417, *p* < 0.001**	***r* = –0.415, *p* < 0.001**

*Significant correlations are represented in bold.*

In the CRYPTO-AF cohort, AF was detected in 50 subjects. No patient had AF when blood was collected. Cryptogenic stroke patients with AF had higher levels of GDF-15, IL-6, Troponin-T, Ang-2, BMP-10, DKK-3, and total NT-proBNP, in comparison to those without AF (Table 1 and Figure 2). In this cohort, information about AF duration was available for 38 patients. Median number of AF episodes was 10 (3–34.25) and median longest AF episode was 626.12 min (130.24–1,409.07 min). The number of episodes correlated with the level of Ang-2, while the duration of the longest episode also correlated with GDF-15, Troponin-T, IL-6, and total NT-proBNP (Table 2). The correlations between biomarkers and left atrial function were reported in Table 2.

Total NT-proBNP, Ang-2, and Troponin-T were increased in both cohorts (Table 1 and Figure 2). When comparing the two cohorts, some biomarkers presented differential concentration: IL-6, DKK-3, ESM-1, Troponin-T, and total NT-proBNP were higher in the CRYPTO-AF cohort compared to AFRICAT cohort, while GDF-15, FGF-23, and IGFBP-7 presented lower concentrations. These differences were only significant between the AF group for DKK-3 and between the no AF group for GDF-15, Troponin-T, and total NT-proBNP (Figure 2). As the main difference between the two cohorts, apart from the stroke itsef was the presence of hypertension and diabetes in all the patients included in AFRICAT cohort, the association of these pathologies with all the biomarkers was tested in the CRYPTO-AF cohort. GDF-15 (*p* < 0.001), Troponin-T (*p* = 0.002), FGF-23 (*p* = 0.006), and IGFBP-7 (*p* = 0.018) abundance was higher in patients with hypertension. Moreover, patients with diabetes had higher concentrations of GDF-15 (*p* = 0.017) and Troponin-T (*p* = 0.013), and lower concentrations of ESM-1 (*p* = 0.048).

Some biomarkers presented significant correlations with other biomarkers and age (Supplementary Figure 2).

Sensitivity analyses were conducted excluding outliers and results were similar (Supplementary Table 1). The only remarkable differences were in the univariate analysis of the CRYPTO-AF cohort, in which IGFBP7 was significant (*p* = 0.030) and IL-6 was no longer significant (*p* = 0.063).

### Biomarker Combinations

#### Asymptomatic High-Risk Subjects

**FIGURE 3:**
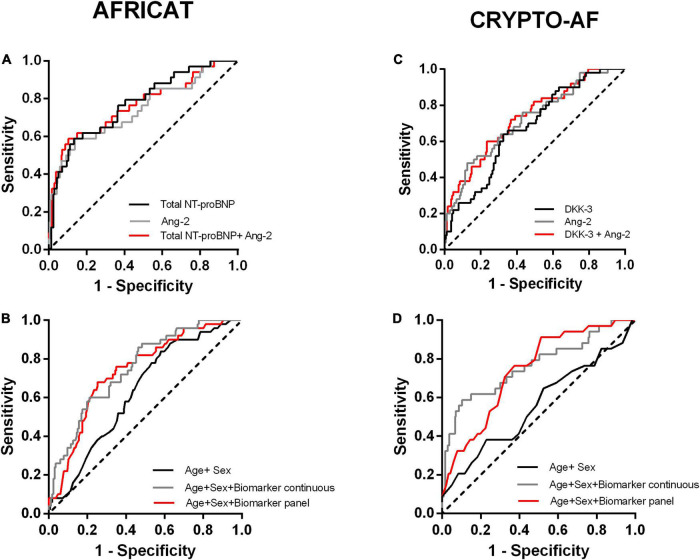
Receiver operating characteristic (ROC) curves of the constructed models for the two cohorts. **(A, B)** Performance of the models constructed for the AFRICAT cohort. **(C, D)** Performance of the models constructed for the CRYPTO-AF cohort. **(A, C)** ROC curves including Ang-2,Total NT-proBNP, or DKK-3 alone, and combined as continuous variables for each cohort. **(B, D)** ROC curves for the clinical model alone (Age + Sex) and in combination with the biomarkers as continuous variables or dichotomized according to the panel with the best accuracy.

Angiopoietin-2 and Total NT-proBNP were selected to be included as explanatory variables in the logistic regression model by backward selection. The model including both biomarkers together with age and sex had an AUC of 0.764 (0.665–0.863), which was better than the performance of Ang-2 alone [AUC = 0.741 (0.640–0.843)] (DeLong = 0.044), but similar to the one of total NT-proBNP alone [AUC = 0.771 (0.683–0.860), *p* < 0.001] (DeLong = 0.731; Figure 3).

The model including the biomarkers showed a higher accuracy in terms of area under the curve than the clinical model [AUC 0.559 (0.445–0.673)] (Figure 3 and Table 3). Similar results were obtained when we adjusted by other cardiovascular factors (Supplementary Table 3).

**TABLE 3:** Logistic regression analyses and additional predictive value of blood biomarkers as continuous variables in the AFRICAT and the CRYPTO-AF cohort.

	AFRICAT		CRYPTO-AF	
	Clinical model (Age + Sex)	Clinical model (Age + Sex) +Biomarkers_continuous	Clinical model (Age + Sex)	Clinical model (Age + Sex) +Biomarkers_continuous
**Logistic regression, OR (95% CI)**				
Age	0.982 (0.929–1.037), *p* = 0.517	0.933 (0.837–1.041), *p* = 0.221	1.044 (1.010–1.080), *p* = 0.010	1.036 (0.999–1.073), *p* = 0.056
Sex	0.962 (0.739–1.252), *p* = 0.773	0.667 (0.342–1.301), *p* = 0.235	0.955 (0.574–1.590), *p* = 0.861	0.977 (0.573–1.666), *p* = 0.931
Ang2 (ng/ml)	–	1.635 (1.216–2.198), *p* = 0.001	–	1.451 (1.160–1.814), *p* = 0.001
TotalNTproBNP (pg/ml)	–	1.000 (1.000–1.000), *p* = 0.030	–	–
DKK-3 (ng/ml)	–	–	–	1.016 (0.997–1.036), *p* = 0.087
IDI statistics				
Total IDI (95% CI)		16.8% (8.32–25.4%)		9.87% (4.95–14.79%)
*P*-value		0.0001		0.000084
**ROC curve**				
AUC	0.559 (0.445–0.673)	0.764 (0.665–0.863)	0.631 (0.550–0.712)	0.733 (0.654–0.813)
DeLongTest		*p* = 0.0012		*p* = 0.0163

*De Long test compared the performance of the clinical model with the biomarkers, and the clinical model alone in each cohort.*

*AUC indicates area under the curve; OR, odds ratio; and ROC, receiver operator characteristic.*

**FIGURE 4:**
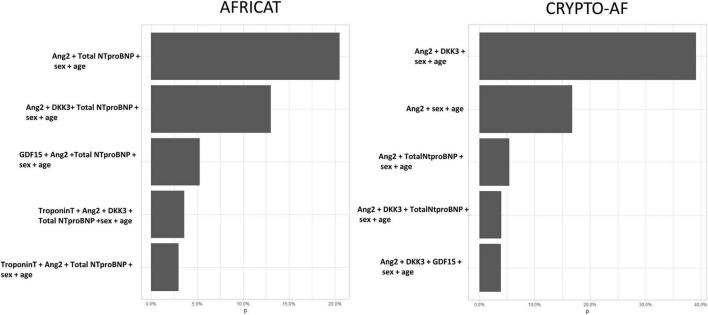
Five most frequent biomarker combinations after 1,000 stepwise logistic regression boostrap iterations. *X* axis represents de percentage of boostrap iterations in which the combination was selected. In total, 150 combinations were selected in the AFRICAT cohort, and 72 in the CRYPTO-AF cohort.

After 1,000 boostrap iterations the combination of Total NT-proBNP and Ang-2 was selected in 20.5% of the times. Other frequent combinations included are shown in Figure 4.

A panel including Ang-2 > 1.73 ng/ml and Total NT-proBNP > 665.86 pg/mL was selected by the PanelomiX software as the most accurate (AUC = 0.7331) for the detection of AF in asymptomatic patients. The panel was positive when the two markers were above the cut-off (sensitivity, 76.5%; specificity, 70.8%). Alternatively, a panel including Ang-2 > 2.613 ng/ml and Total NT-proBNP > 632.65 pg/mL, positive when one of the biomarkers was above the cut-off had a sensitivity of 91.2% and a specificity of 47.5% (AUC = 0.7539). The inclusion of the biomarkers as panels to the logistic regression models resulted in an AUC of 0.778 (0.696–0.858) and 0.740 (0.657–0.823), respectively, (Figure 3 and Supplementary Table 3). Multicollinearity was not detected in any of the models.

##### Comparison to Biomarkers Previously Measured in the Cohort (NT-ProBNP)

In the AFRICAT cohort, NT-proBNP (Roche Diagnostics) had been previously measured (6). The pre-commercial total NT-proBNP assay, measuring all, glycosylated and non-glycosylated, forms of NT-proBNP had similar AUC [0.775 (0.69–0.86)] in comparison to conventional NT-proBNP [0.771 (0.69–86)] in the present cohort (De Long = 0.807), although conventional NT-proBNP had a better discriminatory capacity [IDI = 4.78%[1.40–8.17%)]. Both biomarkers presented a strong correlation (*r* = 0.944, *p* < 0.001).

If conventional NT-proBNP was also included in the backward stepwise model, the combination of Ang-2 and conventional NT-proBNP would be selected as the best combination. In fact, the accuracy of the model including these biomarkers together with age and sex was similar [AUC = 0.757 (0.655–0.859)] to the one obtained with the combination of Total NT-proBNP and Ang-2 (DeLong = 0.444), but the discrimination capacity was better [IDI = 1.96% (0.15–3.77%)].

#### Cryptogenic Stroke Patients

Ang-2 and DKK3 were selected by backward selection to be included as explanatory variables of post-stroke AF. The model including both biomarkers together with age and sex had an AUC of 0.733 (0.654–0.813), which is better than the performance of DKK-3 alone [AUC = 0.677 (0.595–0.757)] (DeLong = 0.0.029), but similar to the performance of Ang-2 alone [AUC = 0.719 (0.6374–0.802)] (DeLong = 0.462; Figure 3).

The model including the biomarkers showed a higher accuracy in terms of area under the curve than the clinical model [AUC 0.631 (0.550–0.712)] (Figure 3 and Table 3). Similar results were obtained when we adjusted by other cardiovascular factors (Supplementary Table 3).

After 1,000 boostrap iterations the combination of Total NT-proBNP and Ang-2 was selected in 39.1% of the times. Other frequent combinations included Total Nt-proBNP (Figure 4).

A panel including Ang-2 > 1.52 ng/ml and DKK-3 > 54.07 ng/mL was selected by the PanelomiX software as the most accurate (AUC = 0.695) for the diagnosis of AF after stroke. The panel was positive when the two markers were above the cut-off (sensitivity, 76%; specificity, 64.1%). Alternatively, a panel including Ang-2 > 1.41 ng/ml and DKK-3 > 43.52 ng/mL, also positive when both biomarkers were above the cut-off, had a sensitivity of 90% and specificity of 40.1% (AUC = 0.647). The addition of the biomarker panels to the logistic regression models resulted in an AUC of 0.738 (0.662–0.813) and 0.717 (0.643–0.792), respectively, (Figure 3 and Supplementary Table 3). Multicollinearity was not detected in any of the models.

##### Comparison to Biomarkers Previously Measured in the Cohort (BNP and NT-ProBNP)

In the CRYPTO-AF cohort, NT-proBNP (Roche Diagnostics) and BNP (Siemens Healthcare) had been previously measured (5).

The pre-commercial total NT-proBNP assay had higher AUC [0.718 (0.64–0.79)] than conventional NT-proBNP [0.668 (0.589–0.747)] (DeLong = 0.002; IDI = 1.67% [0.509–2.831%]) but similar to BNP [0.722 (0.645–0.799); DeLong = 0.82; IDI = –0.044% (–1.64-1.55%)] in the present cohort. Conventional NT-proBNP (*r* = 0.939, *p* < 0.001) and BNP (*r* = 0.839, *p* < 0.001) presented a strong correlation with the pre-commercial assay. If BNP and conventional NT-proBNP were also included in the backward stepwise model, the combination of DKK-3 and Ang-2 would be selected as the best combination.

## Discussion

In the present study, 10 candidate AF blood-biomarkers of various biological pathways described for AF pathophysiology have been measured in blood samples from 492 patients of two cohorts. The two selected study populations included (high-risk asymptomatic individuals and cryptogenic stroke patients) would benefit from AF diagnosis markers.

As a result, four proteins (Troponin-T, Ang-2, ESM-1, and Total NT-proBNP) showed increased levels in the asymptomatic population with AF. Also, we must highlight that the tendency for most of the markers measured was to increase in those groups where “AF burden” was higher (i.e., AF diagnosed by several methodologies), while no biomarker was able to detect paroxysmal AF in our cohort. Complementary to the biomarker abundance distribution among groups, correlations between AF burden and biomarkers were calculated, but only two biomarkers showed significant correlations (total NT-proBNP and FGF-23). However, we should highlight that the lack of registers of some of the patients of the MH + ECG + group, which would have probably presented the highest AF burden measures, and in which monitoring was optional, reduced the statistical power of these correlations. These results are supported by other studies in which biomarkers like NT-proBNP presented higher values according to the AF burden (20). We may hypothesize that the biomarker profiles observed reflected a more “advanced” stage of the disease, with more pathophysiological changes, such as atrial stretch and dilatation (associated with NT-proBNP release), taking place in patients with established AF, in comparison to patients with paroxysmal AF. According to a published meta-analysis, stroke risk is lower in paroxysmal AF, but still higher than in patients with sinus rhythm (9). Therefore, the difficulty of the proteins tested to identify paroxysmal cases reduces its potential as screening biomarkers in asymptomatic populations. Conversely, almost all the proteins tested presented significantly elevated levels in patients with AF detected after a cryptogenic stroke, underlining the usefulness of biomarkers in this setting. Also, similarly to what we found in asymptomatic individuals, NT-proBNP, together with other biomarkers, such as Ang-2, correlated with AF burden measures.

In the AFRICAT cohort the combination of Ang-2 and total NT-proBNP showed the best results, while in the CRYPTOFA cohort the combination of Ang-2 with DKK3 was the one selected, but also the combination of Ang-2 and Total NT-proBNP was between the most frequent combinations after a bootstrapping strategy. These biomarkers represented different pathways affected by AF. Based on its biology, NT-proBNP and DKK3 showed atrial changes whileAng-2 is a marker of vascular damage, suggesting that both atrial and vascular changes are present in AF pathophysiology. On the one hand, total NT-proBNP is released by the cardiomyocytes in response to pressure or volume overload indicating cardiac dysfunction (21). NT-proBNP had been previously presented as one of the most promising AF biomarkers (9, 22), and even validated in the two cohorts presented here (5, 6). Interestingly, this molecule has nine known O-glycosylation sites, some of them located in the central region, where most of the commercial immunoassays are directed, making the molecule almost “invisible” when glycosylated. Consequently, most assays only detect a subfraction of endogenous NT-proBNP (23) but in the present study, the total NT-proBNP assay detected both glycosylated and non-glycosylated NT-proBNP forms. On the other hand, DKK3, a member of the dickkopf family, is a cardiac-enriched protein that plays an important role in heart development and presents cardioprotective functions as a negative regulator of pressure overload-induced cardiac hypertrophy (24). In AF, increased DKK-3 levels have been previously reported, in atrial and blood samples (15, 25).

Next to such atrial changes (reflected by NT-proBNP, or DKK3), Ang-2, a marker of endothelial dysfunction, suggests that there is a pronounced vascular remodeling in AF patients, which could add a further causality to the increased thromboembolic risk in AF next to the blood stasis in the atrium generally described as major stroke risk. The contribution of endothelial injury to the stroke risk is further substantiated by the observation that restoration of the rhythm by ablation or antiarrhythmic drugs is generally not normalizing the stroke risk (26). From the few studies that had explored Ang-2 in AF individuals, Lip et al. found significantly higher levels of this protein together with other angiogenic factors, and platelets in AF patients compared to healthy controls, hypothesizing the role of this protein in the AF prothrombotic state (13, 14). Ang-2 had also been recently associated with the risk of incident AF in the Framingham cohort (12). Also, our results complement the ones of the study of Staszewsky et al. (25), in which a panel with similar biomarkers was tested in patients with history of AF and total NT-proBNP and Ang-2 were significantly associated with ongoing AF together with BMP-10. In their study only total NT-proBNP predicted recurrent AF.

Although in our study the combination of two biomarkers (Ang-2 and Total NT-proBNP, or Ang-2 and DKK-3) optimized the AIC of the model and was selected by a backward stepwise strategy, when we compared their combined performance with the DeLong test vs the performance of each biomarker alone, we obtained similar results. Therefore, the combination of both biomarkers should be tested in other studies to ensure their additive results. Moreover, the presented cut-offs, selected automatically by the PanelomiX algorithm might serve as a reference for replication purposes in further studies. In the present study we only explored cut-offs optimizing accuracy and sensitivity. The combination of both biomarkers, if confirmed in future studies, might be used in clinical practice to screen for AF and a high sensitivity is aimed for screening purposes. Then, in those cases with elevated markers, intensive monitoring periods, and anticoagulation, in case AF is confirmed, would be recommended as primary or secondary stroke prevention strategies. Also, we must highlight that all the biomarkers were measured by automatic immunoassays which makes the translation into the clinical practice easier.

According to our results, total NT-proBNP correlated with other natriuretic peptides and their performance was similar. Also, when combined with Ang-2 and basic clinical variables, the natriuretic peptides previously tested presented similar results.

One of the strengths of the current study is that we measured the same proteins in two cohorts electrocardiographically monitored during 1 month, with the main difference that one presented a previous stroke and the other did not. Therefore, biomarker changes between cohorts may be influenced by the index stroke. In line with our results, IL-6, had been described to increase after stroke as part of the post-stroke inflammatory response with a maximum increase on day 3 (27). Similarly, the level of NT-proBNP was known to increase after stroke within the first 2 days (28). In our study, this biomarker only showed differences between cohorts in the group without AF, and we may hypothesize that the effect of stroke on NT-proBNP is not so important in AF individuals as the biomarker is already increased in those patients. Also, previous results showed an increase in ESM-1 after stroke, although, in contrast with our study, the biomarker was tested in large-artery atherosclerotic stroke patients (29). Moreover, differences between cohorts may be due to their baseline differences. Essentially, all the AFRICAT patients had hypertension and diabetes whereas the percentage of these pathologies was lower in the CRYPTO-AF cohort. Therefore, biomarkers with higher levels in the first study may be associated with these pathologies. Our results and previous publications supported this hypothesis for GDF-15, FGF-23, and IGFBP-7 (30–32). Finally, another explanation is that patients with AF detected after stroke may have an increased thromboembolic risk and/or a higher degree of atrial substrate underlying AF, affecting the expression and release of several proteins. This hypothesis could explain the rise of biomarker levels in the AF group of the CRYPTO-AF cohort in comparison to the same group of the AFRICAT cohort and could be the reason why more biomarkers were increased in AF in this cohort in comparison to the general population. In particular, this could explain why DKK-3 was associated with AF in the CRYPTOFA cohort, while this biomarker did not show differences in the AFRICAT cohort. Furthermore, and supporting this hypothesis, most of the biomarkers tested correlate either with LAS or with LAVI, measures of enlargement, and deformability of the atria, that evaluate the atrial substrate underlying AF (33). All the differences explained and their possible influence in biomarker levels prevented a common analysis pooling both cohorts. Also, the AF detection rate was higher in the CRYPO-AF cohort (22.9%) than the AFRICAT cohort (12.4%). This is in line with previous studies in which prevalence rates of AF were higher in studies including patients after cryptogenic strokes, than in studies including primary care patients (3, 34).

Samples in both studies were taken in a single time-point, just before the monitoring period started. However, longitudinal changes in these markers may be useful to monitor the degree of atrial substrate and search for AF (34). Therefore, the study of serial samples should be explored in future studies as well their implementation into AI-based personalized prevention strategies and medical decisions.

The present study has several limitations that should be mentioned. First, patients were only monitored for 28 days. Longer monitoring periods would probably have detected a higher amount of AF patients (35), which were classified in the no AF group adding background noise to the analysis. Second, as two different cohorts were included, some registered variables and protocols slightly differed. This is the case of AF burden measurement, which in the AFRICAT cohort was calculated as a percentage and in the CRYPTO-AF cohort as the number of episodes and the duration of the longest one, making the comparison difficult. Another limitation was the lack of echocardiographic information in the AFRICAT cohort. Also, we did not correct the models with other variables beyond age and sex as these were the only variables measured in both cohorts that did not present missing information. As a result, the AUC of the models presented was moderate (between 0.7 and 0.8) and the addition of other clinical variables or biomarkers may improve their performance. Another limitation is that biomarkers were selected from the literature and we can not discard that other non-tested proteins could perform better. Finally, the number of AF patients was limited and some associations may have been missed. Validation studies in larger cohorts are needed.

## Conclusion

Blood-biomarkers, in particular, total NT-proBNP, DKK-3, and Ang-2, were increased in AF patients and reflect two mechanistically different pathways involved in AF pathophysiology (AF stretch and vascular changes). They could be useful in AF screening strategies in the primary care setting and also for searching AF after cryptogenic stroke.

## Data Availability Statement

The raw data supporting the conclusions of this article will be made available by the authors, without undue reservation.

## Ethics Statement

This study was reviewed and approved by Comité de ética de investigación con medicamentos y comisión de proyectos de investigación del hospital universitari Vall d’Hebrón [PR(AG)459/2020]. The patients/participants provided their written informed consent to participate in each respective study (AFRICAT and CRYPTO-AF).

## Author Contributions

EP: samples handling, statistical analysis of the data, and drafted the manuscript. AB, JP, JJ, MR, MD, JFA, JC, SP-S, FM, RT, JC-E, JB-O, DR, FG-L, MÁ, and CM: patient recruitment. JF-P, JA, DG, and AlP: Holter-ECG readings. TG-A: Ecocardiographic measurements. AnP: samples handling. JM and CM: obtained funding and developed the protocol for the studies included in the present manuscript. All authors have critically reviewed the manuscript and approved the final version.

## Conflict of Interest

Neurovascular research laboratory, Vall d’Hebron Institute of Research (VHIR) received institutional research support from Roche Diagnostics that funded part of the present study. JM and AB have participated in scientific advisory boards organized by ROCHE on the topic of “New biomarkers in Atrial Fibrillation.”

## Publisher’s Note

All claims expressed in this article are solely those of the authors and do not necessarily represent those of their affiliated organizations, or those of the publisher, the editors and the reviewers. Any product that may be evaluated in this article, or claim that may be made by its manufacturer, is not guaranteed or endorsed by the publisher.
