# Supplementary material for: Blood-Based Biomarkers to Search for Atrial Fibrillation in High-Risk Asymptomatic Individuals and Cryptogenic Stroke Patients
Source: Front Cardiovasc Med. 2022 Jul 4;9:908053. doi: 10.3389/fcvm.2022.908053 (PMC9289129; doi:10.3389/fcvm.2022.908053)
Supplement: Supplementary file 1 [file Data_Sheet_1.PDF]

Supplementary Material

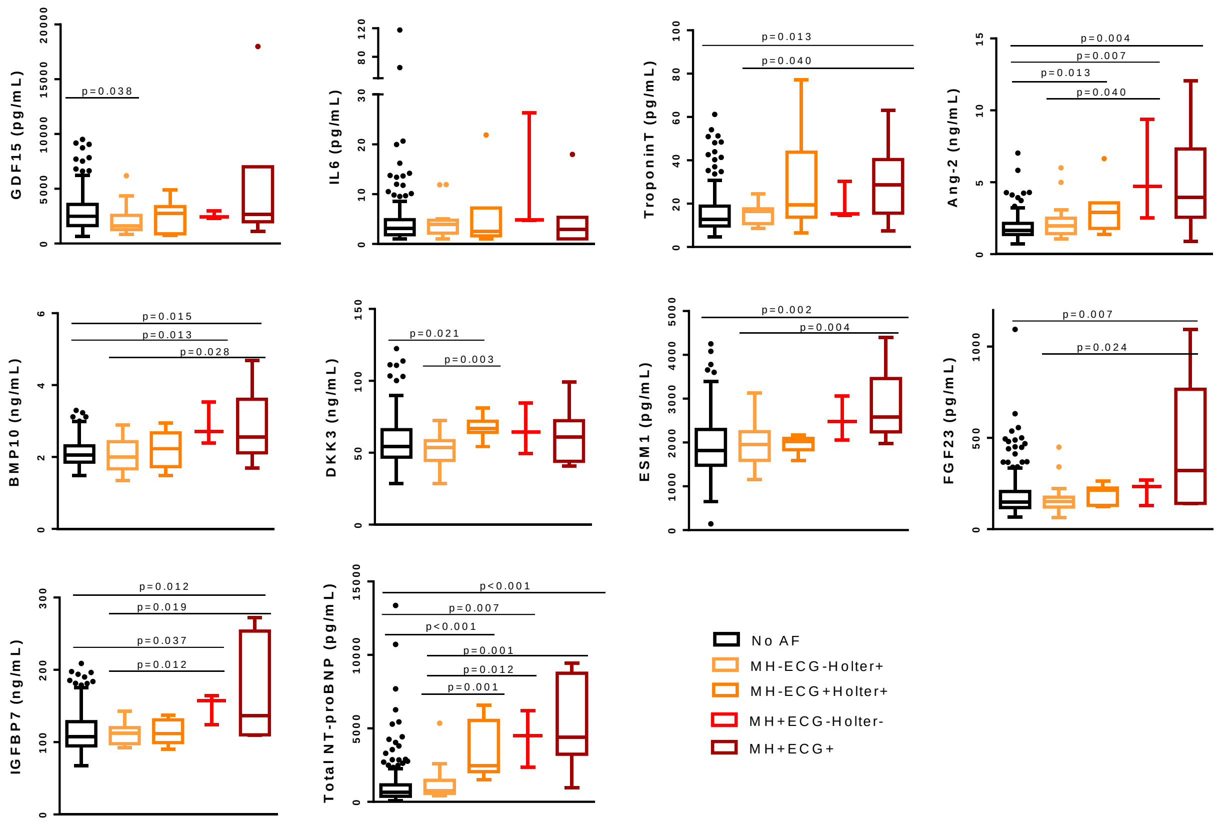

Supplemental Figure 1: Plasma biomarker distributions in the AFRICAT cohort. Boxplots comparing AF groups and no AF patients.

Boxes extend from the 25th to 75th percentiles. The line in the middle is plotted as the median. Whiskers are drawn according to Tukey methodology (±1.5 IQR) and larger values are plotted as individual points. P-values were not corrected by multiple comparisons.

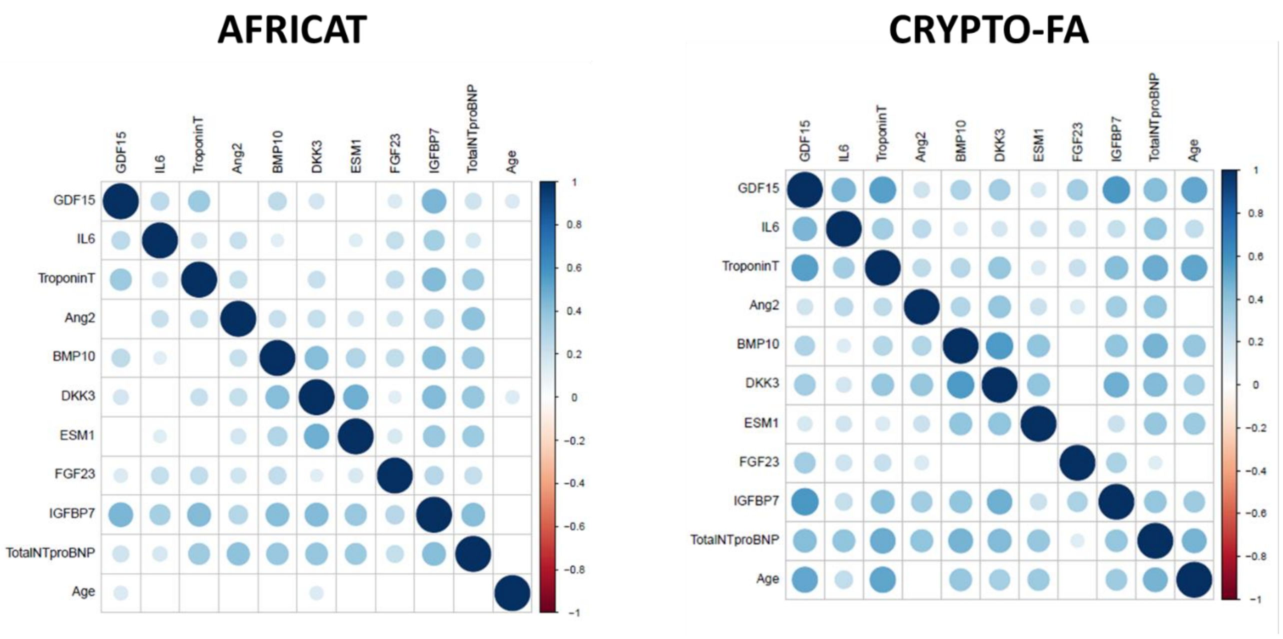

Supplemental Figure 2: Correlation matrix between the proteins measured and age.

Only significant correlations are showed. The colors and the size of the points indicate the magnitude of the correlation coefficient.

Supplemental Table 1: Sensitivity univariate analysis excluding outliers.

	AFRICAT			CRYPTO-AF		
	**AF**	**No AF**	**P-value**	**AF**	**No AF**	**P-value**
**GDF-15 (pg/ml)**	2298 (1325.5-3077.00) n=33	2439 (1623.5-3542.5) n=236	0.295	1896.5 (1521.5-2659.5) n=50	1623.5 (1154.25-2256.25) n=164	0.008
**IL-6 (pg/ml)**	3.64 (2.07-4.91) n=34	3.13 (1.85-4.84) n=238	0.292	9.95 (4.45-16.70) n=48	7.12 (2.74-14.66) n=165	0.063
**TroponinT (pg/ml)**	16.76 (11.62-21.87) n=31	12.78 (9.68-18.53) n=233	0.025	17.18 (14.23-27.12) n=49	15.11 (10.26-20.95) n=167	0.004
**Ang-2 (ng/ml)**	2.17 (1.65-3.00) n=29	1.67 (1.37-2.11) n=238	0.003	2.42 (1.60-3.31) n=47	1.68 (1.31-2.29) n=167	<0.001
**BMP-10 (ng/ml)**	2.23 (1.73-2.55) n=31	2.06 (1.86-2.31) n=240	0.331	2.24 (1.92-2.50) n=49	2.05 (1.78-2.32) n=165	0.014
**DKK3 (ng/ml)**	55.84 (51.05-66.29) n=34	53.80 (46.78-65.28) n=234	0.280	62.02 (55.51-76.23) n=48	55.18 (49.22-64.95) n=166	0.002
**ESM-1 (pg/ml)**	2087 (1828.45-2368.95) n=34	1813.90 (1480.37-2281.25) n=234	0.018	2679.75 (1969.32-3246.7) n=48	2247.5 (1837.0-3148.05) n=165	0.165
**FGF-23 (ng/ml)**	166.44 (129.53-244.63) n=33	148.80 (118.39-206.60) n=240	0.120	134.04 (110.00-204.46) n=50	134.53 (101.97-183.31) n=167	0.538
**IGFBP-7 (ng/ml)**	115.18 (99.61-124.58) n=32	107.53 (94.91-128.29) n=240	0.233	104.42 (85.50-116.84) n=50	94.50 (8.90-107.93) n=165	0.030
**Total NT-proBNP (pg/ml)**	1492.5 (743.35-3246.5) n=31	630.60 (374.34-1153.10) n=236	<0.001	2076.20 (3520.75-1238.15) n=49	859.27 (412.4-1734.22) n=164	<0.001

Values are reported as median (IQR). Sample size for each biomarker and group without outliers is indicated in the sample

Supplemental Table 2: Logistic Regression Analyses and Additional Predictive Value of the Blood Biomarkers panels selected by PanelomiX in the AFRICAT and the CRYPTO-AF cohort

	AFRICAT			CRYPTO-AF		
	Clinical model (Age+Sex)	Clinical model (Age+ Sex) + Biomarkers_panel_accuracy	Clinical model (Age+ Sex)+ Biomarkers_panel_sensitivity	Clinical model (Age+Sex)	Clinical model (Age+ Sex) + Biomarkers_panel_accuracy	Clinical model (Age+ Sex)+ Biomarkers_panel_sensitivity
**Logistic regression, OR (95% CI)**						
**Age**	0.982 (0.929-1.037), p=0.517	0.924 (0.825-1.034), p=0.172	0.920 (0.822-1.029), P=0.145	1.044 (1.010-1.080), p=0.010	1.036 (0.988-1.077), p=0.065	1.045 (1.002-1.091), p=0.012
**Sex**	0.962 (0.739-1.252), p=0.773	0.632 (0.308-1.29), p=0.210	0.696 (0.339-1.430), P=0.323	0.955 (0.574-1.590), p=0.861	0.957 (0.538-1.70), p=0.880	0.924 (0.508-1.681), p=0.797
**Biomarker panel**	-	7.246 (3.300-15.910), p<0.001	7.633 (2.726-21.369), p<0.001		3.735 (1.952-7.146), p<0.001	4.159 (1.818-9.517), p=0.007
**IDI statistics**						
**Total IDI (95% CI)**		11.8% (7.54%-16.0%)	8.30% (5.15%-11.4%)		8.33% (5.21%-11.5%)	6.39% (3.99%-8.79%)
**P-value**		$5.20*10^{-8}$	$2.41*10^{-7}$		$1.71*10^{-7}$	$1.80*10^{-7}$
**ROC curve**						
**AUC**	0.559 (0.445-0.673)	0.778 (0.696-0.858)	0.740 (0.657-0.823)	0.631 (0.550-0.712)	0.738 (0.662-0.813)	0.717 (0.643-0.792)
**DeLong Test**		P=0.002	P=0.012		p=0.00219	p=0.0091

The biomarker panels included in the model were those selected by the PanelomiX tool optimizing accuracy or sensitivity. For the AFRICAT cohort the panel optimizing accuracy included Ang-2 >1.73ng/ml and Total NT-proBNP>665.86 pg/mL (positive when the two markers were above the cutoff) and the one optimizing sensitivity included Ang-2 >2.613ng/ml and Total NT-proBNP>632.65 pg/mL (positive when one of the biomarkers was above the cut-off). For the CRYPTO-AF cohort the panel optimizing accuracy included Ang-2 >1.517ng/ml and DKK-3>54.074 ng/mL, and the one optimizing sensitivity included Ang-2 >1.406ng/ml and DKK-3>43.517 ng/mL, considered positive when the two markers were above the cut-off.

IDI and De Long test compared the performance of the clinical model with the biomarker panel, and the clinical model alone in each cohort.

AUC indicates area under the curve; OR, odds ratio; and ROC, receiver operator characteristics

Supplemental Table 3: Logistic Regression Analyses and Additional Predictive Value of the Blood Biomarkers panels selected by PanelomiX in the AFRICAT and the CRYPTO-AF cohort

	AFRICAT		CRYPTO-AF	
	Clinical model	Clinical model + Biomarkers_continuous	Clinical model	Clinical model + Biomarkers_continuous
**Logistic regression, OR (95% CI)**				
**Age**	0.981 (0.928-1.037), p=0.510	0.933 (0.837-1.041), p=0.217	1.036 (1.005-1.068), p=0.022	1.034 (0.998-1.070), p=0.061
**Sex**	0.962 (0.736-1.257), p=0.778	0.687 (0.354-1.333), p=0.2668	1.001 (0.649-1.567), p=0.969	1.005 (0.609-1.661), p=0.983
**Hypertension**	-	-	0.945 (0.587-1.522), p=0.816	0.996 (0.572-1.736), p=0.989
**Diabetes**	-	-	1.012 (0.633-1.614), p=0.961	1.034 (0.603-1.775), p=0.902
**Ischaemic cardiopathy**	1.051 (0.798-1.384), p=0.721	1.215 (0.584-2.529), p=0.602	0.974 (0.579-1.640), p=0.921	0.916 (0.496-1.691), p=0.778
**Heart failure***	1.021 (0.769-1.355), p=0.886	0.690 (0.270-1.760), p=0.438	0.986 (0.569-1.709), p=0.961	1.007 (0.518-1.960), p=0.982
**Ang2 (ng/ml)**	-	1.637 (1.219-2.199), p=0.010	-	1.365 (1.107-1.683), p=0.0036
**TotalNTproBNP (pg/ml)**	-	1.000 (1.000-1.000), p=0.0271	-	-
**DKK-3 (ng/ml)**		-	-	1.017 (0.998-1.035), p=0.067
**IDI statistics**				
**Total IDI (95% CI)**		16.9% (8.54%-25.3%)		9.5% (4.662%-14.33%)
**P-value**		$7.42*10^{-5}$		0.00012
**ROC curve**				
**AUC**	0.579 (0.464-0.694)	0.782 (0.689-0.876)	0.646 (0.557-0.736)	0.747 (0.663-0.832)
**DeLongTest**		p=0.00098		p= 0.028

De Long test compared the performance of the clinical model with the biomarkers, and the clinical model alone in each cohort.

AUC indicates area under the curve; OR, odds ratio; and ROC, receiver operator characteristic.

The clinical model included age, sex, hypertension, diabetes (only in the CRYPTO-AF cohort as all the patients included in the AFRICAT had both comorbidities), ischemic cardiopathy and heart failure.
